# Impacts to canine dermal microbiota associated with repeated bathing

**DOI:** 10.3389/fvets.2023.1204159

**Published:** 2023-08-09

**Authors:** Dakota Discepolo, Russell Kelley, Adrian Watson, Erin Perry

**Affiliations:** ^1^Department of Animal Science, Food and Nutrition, Southern Illinois University, Carbondale, IL, United States; ^2^Royal Canin USA, Pet Heath Nutrition Center, Lewisburg, OH, United States

**Keywords:** working dog, decontamination, dermal microbiota, canine, bathing

## Abstract

**Introduction:**

Working dogs routinely operate in environmental conditions which may necessitate daily bathing to remove contaminants or soilage. The impacts of frequent or repeated bathing on the canine dermal microbiota are unknown. The objective of this study was to characterize changes in canine dermal microbial populations following repeated daily bathing.

**Methods:**

Labrador retrievers (*n* = 16) were bathed daily using a dilute dish detergent solution (1.6% detergent solution) over the course of 14 days. Dermal microbial DNA was collected *via* sterile swabs (*n* = 142) taken at days 0, 7, 14, 16, 21, 28, 35, 42, and 49 and analyzed for alpha diversity, beta diversity and relative abundance to assess changes in the dermal microbiota *via* 16 s sequencing.

**Results:**

Results indicate that daily bathing significantly increased Shannon diversity, Chao1, and several rare amplicon sequence variants. Although typically reported in highest abundance, relative abundance was decreased in the phyla Actinobacteria, Firmicutes, and Proteobacteria (*p* < 0.05).

**Conclusion:**

Repeated daily bathing with dilute dish detergent significantly reduced normal healthy dermal microbial taxa and created significant changes in the dermal microbiota of canines. Disruption to the canine dermal microbiota may cause negative impacts to canine dermal health and require further investigation.

## 1. Introduction

Current research on the microbiota of the canine dermis has focused on pathologies such as allergies and atopic dermatitis. Previous reports have characterized relative abundance of predominant taxa in both healthy and diseased populations (1–4). Several studies have reported Proteobacteria, Firmicutes, Bacteroidetes, and Actinobacteria in highest abundance (1–4). Many factors may impact the resident dermal microbiota. Atopic dermatitis has been reported to cause significant shifts in microbial composition (1, 5).

Prior efforts aimed at identification of factors impacts dermal microbiota have reported individual variation as the largest contributor to microbial change (4). Other factors include anatomical location of sample and breed. In addition, dietary influence has also been identified as a source of potential impact on dermal microbiota (6). Other factors that may be disruptive to dermal microbiota include topical treatments such as the use of cleansers or detergents.

Exposure to detergents has been reported as a contributing factor leading to dermal microbiota disruption in humans, likely due to altered cutaneous pH (7–10). Dermal irritation has also been associated with altered pH (11, 12). However, the impact of cleansers and detergents on canine dermal microbiota is currently underrepresented in the scientific literature.

Although changes in resident dermal taxa may be of consequence to the canine, there are potential effects for their human counterparts as well. Previous reports have demonstrated that humans in contact with canines develop shared dermal and intestinal microbiota (13–15). Canines and other household pets may act as a fomite and have been reported to carry pathogenic bacteria including *Staphylococcus* (16–19). *S. pseudintermedius*, a resident of the canine dermal microbiota, can colonize human skin potentially causing infection (17). The incidence of infection in humans is not well known as the infection is frequently assumed to be *S. aureus*, which in some cases can also be passed from canine to human (20). Additionally, specific strains of *S. pseudintermedius*, as well as *S. aureus*, are multi drug-resistant which complicates treatment (18, 20). Therefore, factors which increase the abundance of these potential pathogens increase health risks and should be avoided. Pathogens colonizing the dermal microbiome present potential risks to human handlers.

Working canines frequently live and travel in close proximity to their handlers and teams, increasing the potential for spread of pathogenic bacteria should they be colonized. Additionally, working canine management includes frequent use of detergent as part of required decontamination protocols due to the high likelihood of contaminant exposure at deployment areas such as disaster sites (21) or urban environments (22, 23). This recommendation, however, fails to consider that search and rescue (SAR) canines may be deployed to disaster sites for up to fourteen consecutive days requiring daily decontamination(s). Prior assessments from deployed canines responding to the Oso, Washington mudslides reported skin irritation in canines decontaminated daily with a dish detergent within 3 days (24). It is possible that these decontamination procedures may result in shifts of the dermal microbiota which is frequently associated with dermal irritation. The reported symptoms diminished after cessation of detergent usage.

Data is needed to clarify the impacts of daily use of detergent on dermal microbiota as well as its ability to recover once bathing has ceased. Therefore, the objective of this work is to identify the changes in dermal microbiota associated with daily decontamination utilizing a dish detergent and characterize the recovery of the dermal microbiota following cessation of decontamination.

## 2. Materials and methods

### 2.1. Animals and treatments

This research was approved by the Southern Illinois University Institutional Animal Care and Use Committee (15–032) as well as by the Royal Canin ethics committee. Labrador retrievers (*n* = 16) from a research colony were utilized for this study. Labrador retrievers were selected as they are both a popular working and pet breed. Exclusion criteria included use of medications such as antibiotics, history of allergies, and history of dermatological conditions. Canines were housed in their resident kennel environment with two canines per indoor/outdoor run. All canines were up to date on regular monthly parasite control as well as standard vaccinations. Facilities were equipped with an on-site veterinary team in the event of any discomfort or illness in the study population. One canine developed atopic dermatitis on day 14 on the point of hip and in accordance with IACUC guidelines and veterinary team recommendations, was removed from further testing to pursue treatment. Canines were fed a chicken-based diet which was formulated to meet or exceed the NRC requirements with 21% protein and a minimum 10% crude fat. Canines had a minimum of 90 days acclimation to the diet prior to the initiation of the study.

All canines received simulated decontamination once daily for 14 consecutive days. The study protocol was adapted from previously published recommendations working canine decontamination methods (25). Decontamination was carried out by trained technicians with controlled water temperature settings (approximately 36C°), washing order, washing pressure, and rinse times. Decontamination began with total body saturation using a spray nozzle applying water from the base of the neck to the tip of the tail. 16 oz. of dilute dish detergent solution (Dawn^®^ dish detergent, Proctor & Gamble, Cincinnati, OH; diluted 59 mL of detergent to 3.7 L of water) was applied evenly across the shoulders, back, ribcage, chest, abdominal area, and legs. The shoulders, back, left side, right side, chest, and abdominal area of the canine were massaged to a lather. Dorsal and ventral anatomy received a lathering massage for 2 min across each area. The legs were lathered and washed for 30 s each. Each canine was rinsed until no soapy residue remained (approximately 4–5 min). Canines were towel dried using two separate clean towels. The canine coat was left damp but not saturated. All towels were 100% cotton and of identical make and model series (Towelhub^®^, Atlanta, GA).

### 2.2. Data collection and visual assessments

Dermal swabs and visual assessments were collected on days 0, 7, 14, 21, 28, 35, 42, and 49 by a single trained technician. Collection on day 0 occurred prior to the commencement of decontamination. Dermal microbiota swabs were taken from a 3cm^2^ area on the point of the right hip using the Norgen Biotechnology (Ontario, Canada) swab collection and total DNA preservation system utilizing 30 s of skin contact with continuous rotation. In accordance with manufacturer recommendations, collection sites were shaved using a #40 blade (Oster, Boca Raton, FL) following the direction of hair growth prior to each data collection. An additional dermal microbiota collection was added on day 16 to observe changes 48 h following the last decontamination.

Skin health scoring assessments were performed by two trained technicians (see Supplementary material 1). Coat shine, coat condition, back dander and body dander were scored as adapted from previously published works (11, 26, 27). All visual assessments were conducted on a 0–4 (lowest to highest) scale with half point increments acceptable.

### 2.3. Microbiota analysis

Microbial DNA from swabs was extracted at Southern Illinois University utilizing the Norgen Microbiota DNA Isolation Kit (Ontario, Canada). Isolated DNA was submitted for next generation sequencing (Diversigen Inc.) *via* 16S amplicon sequencing pipeline. Low DNA concentrations resulted in the removal of 8 samples. Thus, data reported here include 134 dermal microbial samples. Variables of interest include differences associated with study day, coat shine, coat condition, body dandruff, and back dandruff.

### 2.4. Alpha diversity analysis

Analysis of alpha diversity of canine dermal microbiota was carried out using amplicon sequence variants (ASVs). Rare ASVs, present in less than 10% of the samples, were removed from the ASV table resulting in a total of 1,530 ASVs. Three different alpha diversity metrics, Shannon, Chao1, and Observed ASVs, were calculated from the filtered ASV count table rarefied to the minimum sequencing depth using the vegan package in R. To determine whether daily bathing affects alpha diversity, a linear mixed model including day as a fixed effect and dog identity as a random effect was utilized [alpha diversity ~ day + (1|dog name)]. Additionally, a linear mixed model including day, skin and coat condition scores, and their interaction as fixed effects and dog identity as a random effect [alpha diversity ~ day * variable + (1|dog name)] was constructed and utilized to identify any confounding effect on daily bathing.

### 2.5. Beta diversity analysis

**Figure 1:**
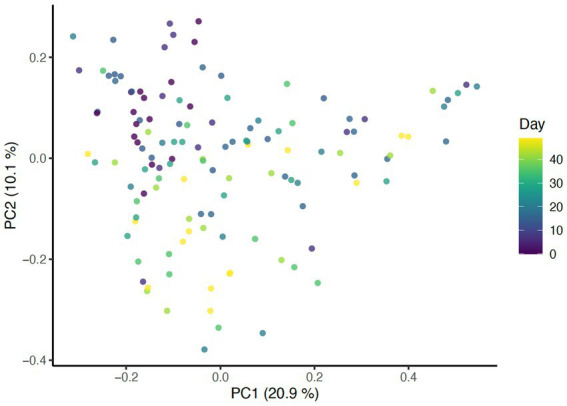
PCoA of Bray Curtis distance colored by study day (*p* = 0.001, *R*^2^ = 0.27).

**Figure 2:**
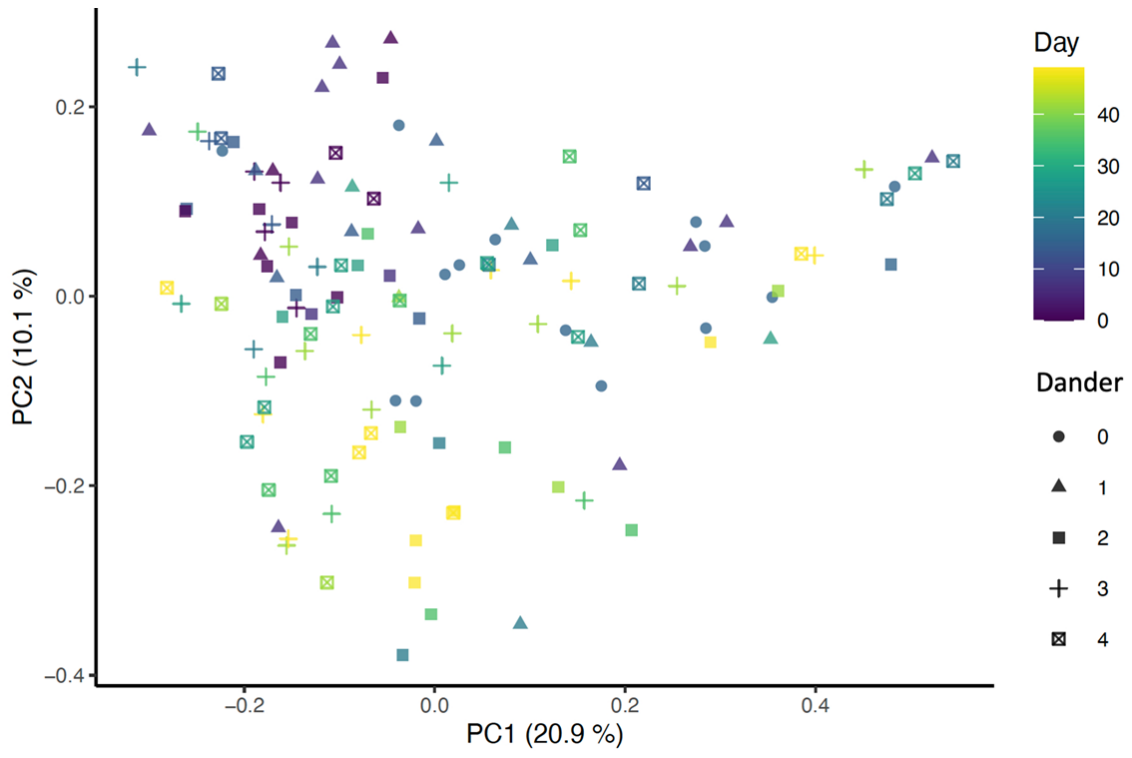
PCoA of Bray-Curtis distance colored by day (*p* = 0.001, *R*^2^ = 0.27) and shaped by dander score (*p* = 0.025, *R*^2^ = 0.0708).

Beta diversity analysis was carried out using three different distance methods: Bray-Curtis, Unweighted UniFrac (considers only presence absence), and Weighted UniFrac (accounts for abundance of taxa). To determine the impact of study day on beta diversity, a Principal Coordinates Analysis (PCoA) plot was generated including all samples colored by days (Figure 1) Additionally, the envfit function from the vegan package in R was used to assess changes to beta diversity across study days as well as effects associated with skin and coat condition scores (Figure 2).

### 2.6. Differential abundance

Differential abundance analyses were performed across all taxonomic levels, including Phylum, Order, Family, Genus, ASV. To account for compositionality of microbiota data, the raw counts of taxa were transformed to Centered Log Ratio (CLR)-transformed abundance distributions using Monte-Carlo (MC) sampling (N = 20 instances) as implemented in the ALDEx2 R package. To assess changes to taxa abundance related to daily bathing, a linear mixed model was created including day as a fixed effect and dog identity as a random effect for each taxon on each MC instance of CLR-transformed abundances: taxon ~ day + (1|dog name). Additionally, a linear mixed model including day, skin and coat condition scores, and their interaction as fixed effects and dog identity as a random effect [taxon ~ day * variable + (1|dog name)] was constructed and utilized to identify any confounding effect on daily bathing.

**Figure 3:**
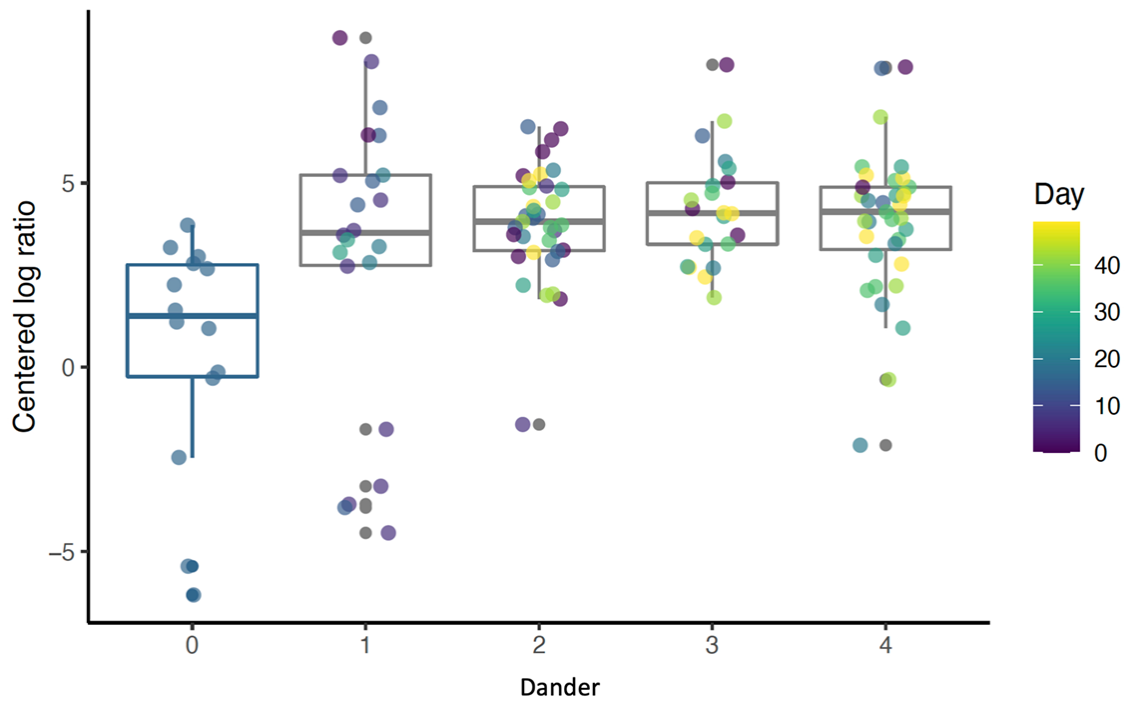
Boxplot showing abundance of Cyanobacteria for different levels of back dander (*p* < 0.001) by day (*p* = 0.69).

Raw value of ps were collected within each MC instance and were corrected for multiple hypothesis testing (testing multiple taxa) using the Benjamini-Hochberg (BH) method. The average expected value of p across all MC instances was calculated for both raw and BH corrected *p*-values and reported as the final result. For any significant taxa, scatter plots of CLR-transformed abundance of taxa over time with an overlaid smooth line from LOESS regression were generated. In addition, if a taxon was significantly associated with a visual assessment, boxplots/strip charts of that variable were created (Figure 3).

## 3. Results

### 3.1. Alpha diversity

**Figure 4:**
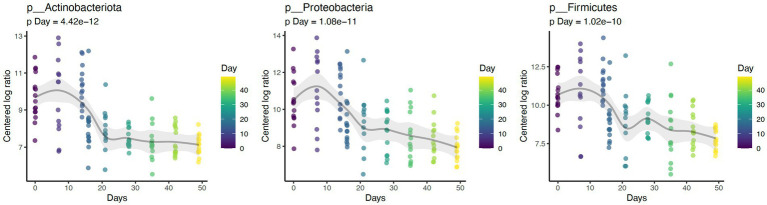
Daily bathing significantly increased Shannon Diversity Index (*p* < 0.001), Chao1 (*p* < 0.001), and number of ASVs observed (*p* < 0.001).

**Figure 5:**
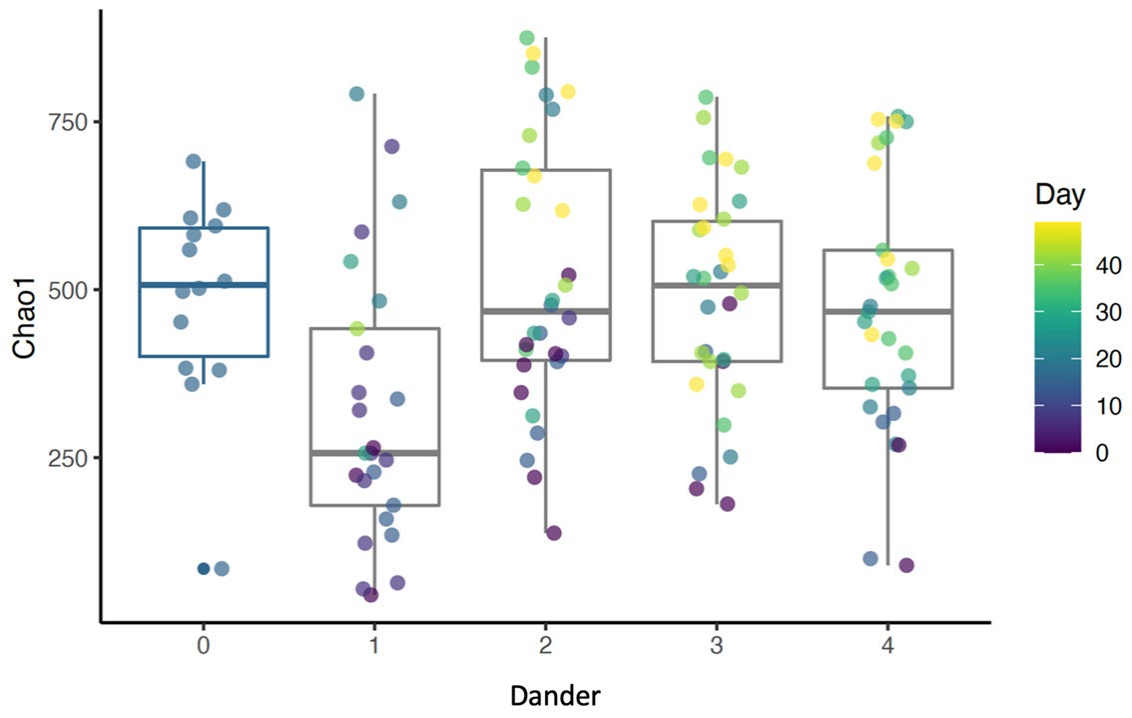
Boxplot showing Chao1 index for different levels of back dander (*p* = 0.0389).

Results show that daily bathing increased Shannon diversity (*p* < 0.001), Chao1 (*p* < 0.001), and number of ASVs observed (*p* < 0.001) throughout the study (Figure 4). Alpha diversity for each measure peaked at day 35. Coat condition impacted Chao1 index (*p* = 0.018). Chao1, which measures the richness of the samples, increased at the lowest coat condition score (score = 1). This reveals an inverse relationship between species richness of the dermal microbiota and coat condition score. Additionally, coat dander impacted Chao 1 (*p* = 0.039) with Chao 1 lowest at dander score of 1 and highest with a score of 2 (Figure 5). As dander scores increased, species richness increased, but as coat condition improved (and scores increased) richness decreased. Coat condition and back dander changes did not interact with the changes associated with study day changes.

### 3.2. Beta diversity

**Figure 6:**
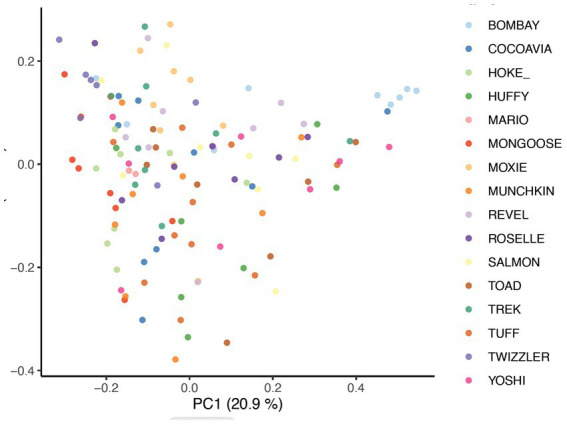
PCoA of Bray-Curtis distance colored by dogs (*p* = 0.001 *R*^2^ = 0.31).

Presence of taxa was impacted by daily bathing as unweighted UniFrac distances were significantly different across days (*p* = 0.001, *R*^2^ = 0.27). As expected, samples from the same dog showed greater similarity when compared to samples from other dogs (*p* = 0.001, *R*^2^ = 0.31) (Figure 6). When taxa abundance was considered, weighted UniFrac distances were unaffected by day (*p* = 0.851, *R*^2^ = 0.00266) but remained clustered by dog (*p* = 0.001, *R*^2^ = 0.345). These data indicate that the abundance of taxa were not impacted by daily bathing, only the presence or absence of specific taxa.

Significant associations between beta diversity metrics and coat condition, coat shine, and back dander were also found indicating each variable contributed to differences between the samples. Using the Bray-Curtis distance, coat condition (*p* = 0.052), coat shine (*p* = 0.004), and back dander (*p* = 0.025) (Figure 2) were associated with changes to the dermal microbiota composition. When controlling for these variables within the statistical model, the difference in the microbial composition between days remained significant (*p* = 0.001, *R*^2^ = 0.27) (Figure 1), Accordingly, Bray-Curtis distance effects appear to be independent of any effects related to study day.

### 3.3. Differential abundance

**Figure 7:**
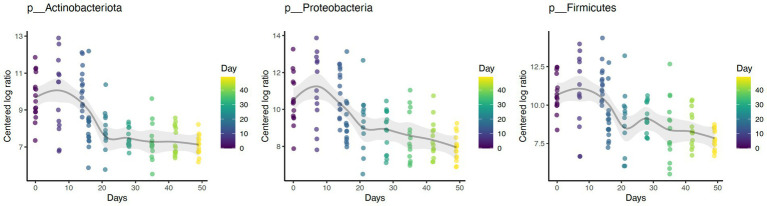
Daily bathing decreased the abundances of taxa Actinobacteria (*p* < 0.001), Firmicutes (*p* < 0.001), and Proteobacteria (*p* < 0.001) at the Phylum level.

**Figure 8:**
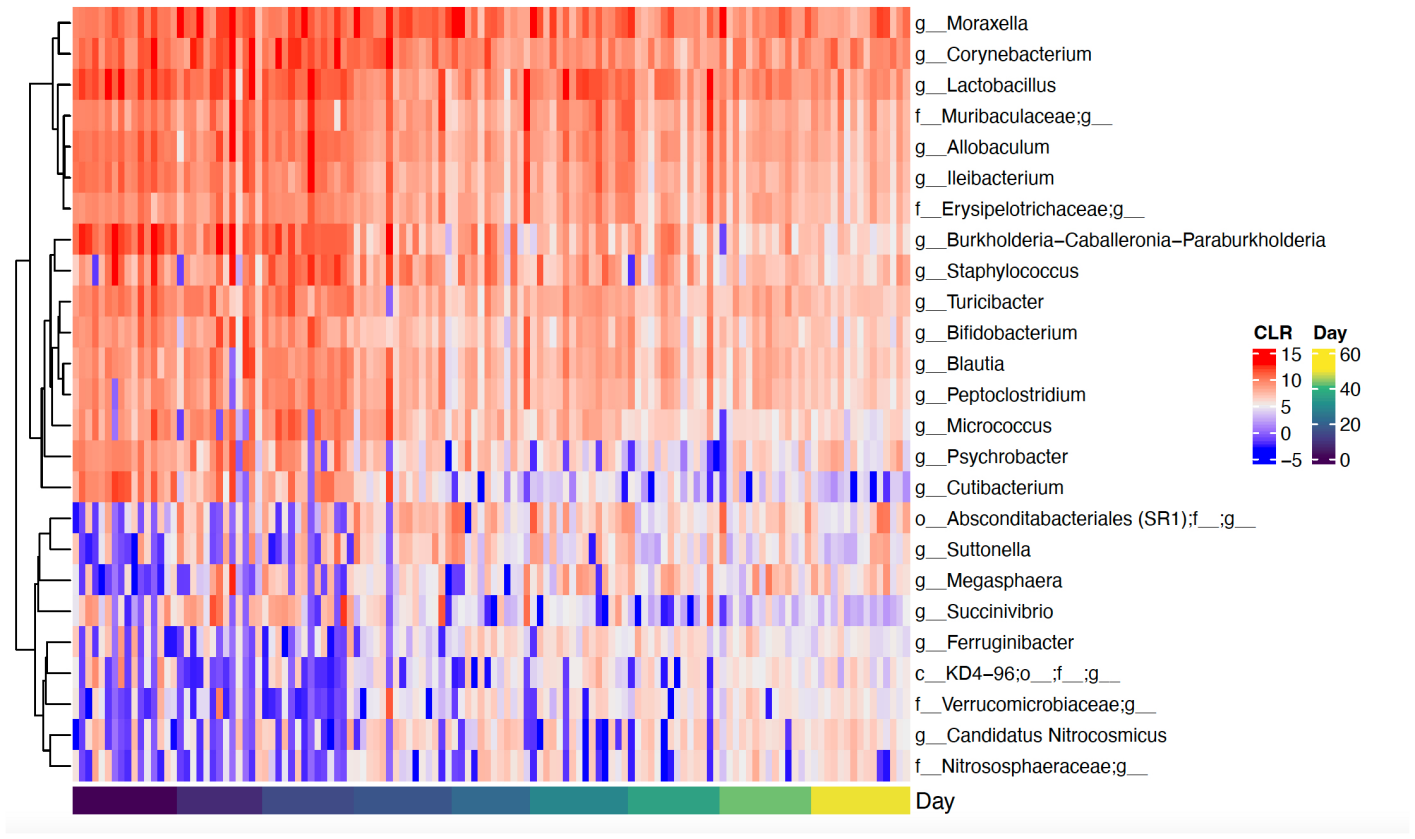
Heatmap showing CLR-transformed abundances of the top 25 most abundant genera that were significantly changed over time. If a genus is unclassified, its higher taxonomic level is shown in the heatmap.

Daily bathing significantly decreased the relative abundance of commonly predominant bacteria including Actinobacteria (*p* < 0.001), Firmicutes (*p* < 0.001), and Proteobacteria (*p* < 0.001) (Figure 7). Additionally, the abundance of 60 taxa at the genus level were significantly changed by daily bathing. The 25 most abundant are shown in a heatmap (Figure 8). Cyanobacteria were significantly related to dander (*p* < 0.001) but not day (*p* = 0.69). Additionally, there was no interaction for changes in Cyanobacteria for day and dander scores (*p* = 0.856). However, it is important to note that abundance of Cyanobacteria increased with increased dander scores (Figure 3).

## 4. Discussion

Improved understanding of the canine dermal microbiota is an important area of research and is a crucial component of canine dermal health. Prior reports of taxa present have focused on organisms associated with disease states such as atopic dermatitis (AD) (5). While prior decontamination recommendations have included the use of dish detergent or an alkaline cleanser for working canines receiving decontamination daily (25), the data presented here challenges those recommendations. Moreover, this is supported by the skin irritation documented following daily decontamination with dish detergent on dogs during a deployment (24). The data presented here provide additional evidence regarding changes to the dermal microbial composition associated with prior recommendations for the use of dish detergent.

Dermal microbiota studies in canines experiencing AD have reported key differences between populations of canines with healthy skin and populations of canines with AD (1, 3, 5, 28). Overall, canines who are healthy and canines with AD have the same general taxa present, but the abundance of that taxa differs by health status (1, 3). Canines from these studies are reported to have predominant phyla including Proteobacteria, Firmicutes, Actinobacteria, Bacteroidetes, and Cyanobacteria level (1, 2, 4). However, contradictory findings were reported in canines suffering from AD with an overall lower number of observed taxa present than healthy dogs (2). These differences may be explained by different coat types, anatomical locations, or varying study population breeds. Nevertheless, the prior studies consistently agree that relative abundance is associated with AD and some key taxa become more abundant when compared to healthy controls.

Similar to prior work, this study identified Proteobacteria, Firmicutes, and Actinobacteriota as the dominant phyla (1, 2, 4). Daily bathing study affected microbial composition as unweighted UniFrac distances were significantly grouped across study day. Furthermore, daily bathing significantly increased Shannon diversity, Chao1, and number of ASVs observed. These findings indicate that species richness as well as abundance of those species differed throughout the study, with greatest impacts observed during bathing the active bathing period. Diversity increased following cessation of bathing and continued throughout the study timeline but failed to return to baseline measures. These results may indicate increased species richness, as well as increased abundance of those species in the dermal microbiome brought on by repeated bathing with dish detergent solution.

It is interesting to note that based on the trends of the presented results, the lowest measures of alpha diversity were observed on day 7. This trend may indicate that the negative impacts to the resident taxa had already occurred after a single week of daily decontamination with very dilute dish detergent solution. However, additional testing is required to further understand the rate at which dermal microbiota becomes compromised and at which it may also recover.

Reported changes in alpha diversity were accompanied by changes in relative abundance of the most predominant dermal microbiota during repetitive daily bathing, with decreases in abundances of these taxa below original baseline measurements throughout the study. These findings, along with the changes in alpha diversity indexes, confirm that serial bathing had lasting impacts to the dermal taxa of the canines. However, it is currently unknown whether these changes are beneficial or not. It is possible that the bathing treatment decreased the abundance of the most dominant taxa allowing the non-resident taxa to utilize more resources and increase in presence. This theory may be supported by decreases in alpha diversity observed at day 7. The predominant phyla in this study (Proteobacteria, Firmicutes, and Actinobacteriota) peaked in abundance at the 7-day mark, after which their abundance decreased, potentially giving rise to increased species richness. Increased richness may not be beneficial in all instances. It is possible that these changes may lead to atopic dermatitis as suggested previously (1–3). Additionally, there is a possible risk associated with increased colonization of zoonotic pathogens present within the environment where the canine may live and or work.

It is unclear from prior studies whether changes in taxa presence and abundance are a result of dermal disease through decreased nutrient availability (water and lipids) or by disrupted dermal barrier integrity. However, authors have concluded that changes in the microbial profile are an indication of changes in dermal health (29, 30). Further work should better identify the relationship between the dermal microbiota, skin disease and barrier disfunction through measurements like trans-epidermal water loss (TEWL) and cutaneous pH (31, 32).

Several studies mentioned above have found that increased abundance in *Staphylococcus* is primarily associated with AD in canines (2, 3, 5). The data presented here demonstrate a significant increase in the abundance of *Staphylococcus* that peaks during the time of serial bathing and then falls below baseline values when daily bathing ceases. This finding may indicate that repetitive, daily bathing of dogs with dish detergent increases the risk for AD or infection. The increased risk may be a result of increased moisture exposure to the skin as well as cleanser effects to dermal pH which may facilitate a preferential environment for *Staphylococcus* to thrive. Although this work does not report beyond genus, should these increased *Staphylococcus* species be potential pathogens such as *S. pseudintermedius* or *S. aureus*, findings could indicate increased risk to handlers and teammates. Future work should identify if the changes in *Stapylococcus* by species associated with repetitive bathing give rise zoonotic pathogens as described.

Prior studies have identified a potential relationship between coat condition scores and dander with skin health (27, 33, 34). The findings presented here provide additional evidence for the relationship between dermal microbiota and coat condition and dander. These findings are novel and offer further evidence that the dermal microbiota plays a crucial role in dermal health. Additionally, these data suggest that the association between visual characteristics and the dermal microbiota based on significant changes in beta diversity using the Bray-Curtis distances. These differences indicate that the visual assessments accounted for a significant amount of the difference between samples when taking into consideration both presence of the taxa as well as the relative abundance. Alpha diversity values of species richness (Chao 1) were significantly different by coat condition in addition to dander. This indicates that as dander becomes more evident (scores increased) species richness decreased, but as coat condition improved (and scores increased) species richness decreased. These are contradictory findings; however, it may be possible that increased lipid production as part of dermal healing may have had an effect on coat condition (35). Lastly, the abundance of several individual taxa changed in conjunction with visual assessments. One example is the change in Cyanobacteria in relation to dander. The abundance of Cyanobacteria increased as dander score increased. The increased abundance of Cyanobacteria is of particular concern due to the cytotoxins (known carcinogens) which are created as secondary metabolites (36). Further research should examine individual taxa changes and potential relationships to changes in coat and dermal health.

It is evident that the serial decontamination of the canines in this study led to lasting changes in the dermal microbiota of the canines. Previous work has demonstrated that the dermal microbiota extends past the surface of the epidermis into the dermis (37), and therefore future work on decontamination and bathing practices should investigate the effects of the dermal microbiota past the surface of the epidermis. Other studies have noted that taxa composition and abundance may be altered by anatomical location. Future work should also sample from various anatomy to identify potential differences (1).

Although the full nature of the relationship between dermal microbiota and dermal health is unknown, the changes in dermal microbiota associated with dandruff occurrence is another finding which appears to support a close relationship. It is also important to note that the cleanser utilized in the decontamination protocols employed for this study is more dilute than that typically used in the field. Dish detergent utilized in the field is typically undiluted and therefore may result in even greater damage. Therefore, future work should seek alternative effective cleansers and/or methods which are approved for veterinary use which may have decreased effects to the dermal barrier and microbiota of the canine.

## 5. Conclusion

Repetitive bathing with highly dilute dish detergent to simulate decontamination practices resulted in significant impacts to the resident microbiota on canine skin. Of these changes is an increase in *Staphylococcus*, which has been previously associated with atopic dermatitis (2, 3, 5) and of which some strains may implicate human health (19, 20). Although the dish detergent utilized for the decontamination protocols was extremely dilute in comparisons to the typical undilute application commonly used in the field, the procedures affected some of the most prominent taxa of the canine dermal microbiota with no recovery to baseline abundance within the 35 days following the final bathing. Trends of these changes in alpha diversity metrics reached a low at day 7, which was only halfway through the bathing series, corresponding to half the time of standard SAR canine deployment. Should these practices be utilized in the field for the full deployment time of 14 days, it is expected that the canines will experience similar if not more severe disruptions to the dermal microbiota. It is additionally possible, that effects be seen after 7 days, however further work is needed to further explore this finding.

Moreover, these data reveal a heretofore unexplored relationship between changes in microbiota and coat condition and dander scores. These associations support that it is possible for changes in the dermal microbiota can be observed visually *via* changes in dander and coat condition assessments.

## 6. Study limitations

As the study was being conducted in Spring of 2020, the COVID 19 pandemic resulted in lack of access to a larger study population due to travel limitations for study technicians. Further limitations include lack of culture-based methods and lack of control population decontaminated with water alone. Future work should test for effects of water-only decontamination, utilize breeds commonly utilized in working disciplines including service and therapy dogs, and include microbial analysis from various anatomical regions to capture a more comprehensive picture of the entire dermal environment and impacts associated with bathing.

## Data availability statement

The data presented in the study can be found in the online repository Figshare. Figshare doi: 10.6084/m9.figshare.23403308 can be found at https://figshare.com/projects/Impacts_to_canine_dermal_microbiota_associated_with_repeated_bathing/169451.

## Ethics statement

The animal study was reviewed and approved by Southern Illinois University Institutional Animal Care and Use Committee (15–032) and the Royal Canin ethics committee.

## Author contributions

DD participated in study design, data collection, data analysis, statistical analysis, and manuscript preparation. RK participated in study design, data collection, statistical analysis, and manuscript review. EP supervised all aspects. AW participated in study design and manuscript preparation and review. All authors contributed to the article and approved the submitted version.

## Funding

All study funding was provided by Royal Canin SAS, 650 Av. de la Petite Camargue, 30470 Aimargues, France.

## Conflict of interest

RK and AW were employed by Royal Canin SAS.

The remaining authors declare that the research was conducted in the absence of any commercial or financial relationships that could be construed as a potential conflict of interest.

## Publisher’s note

All claims expressed in this article are solely those of the authors and do not necessarily represent those of their affiliated organizations, or those of the publisher, the editors and the reviewers. Any product that may be evaluated in this article, or claim that may be made by its manufacturer, is not guaranteed or endorsed by the publisher.
