# Supplementary material for: Impacts to canine dermal microbiota associated with repeated bathing
Source: Front Vet Sci. 2023 Aug 9;10:1204159. doi: 10.3389/fvets.2023.1204159 (PMC10446845; doi:10.3389/fvets.2023.1204159)
Supplement: Supplementary file 1 [file Data_Sheet_1.docx]

**Supplement 1.**  Scoring guide for coat and skin condition to utilize for weekly assessments adapted from prior work (Baranda et al., 2002; Grammer-West et al., 1996; Turner et al., 2012).

**Procedure:**

Prior to assessment, all dogs should be groomed following the according to established grooming procedure (See methods).

**Scoring Guidelines:**

Attributes scored are back dander, dander on the dorsal sides of the body, coat shine, coat softness, and overall coat condition.

A scale of 1 (lowest) to 4 (highest) with half point increments acceptable.

Higher scores indicate decreased skin health.

**Scoring Traits:**

Dander (Back & Sides)

Manually part the hair in several (3-5) areas along the back and in several areas along both the left and right sides of the body. Assess skin flakes (dander) visible at the base of the parted hair and within the fur. (See supplement 2 for scoring guide).

Shine

Observe the coat from several different angles in order to gauge the amount of light reflected. The greater the light reflected, the lower the score. Lighting must remain constant for all evaluations throughout the course of the study for each evaluation.

Softness

Stroke the fur along and against the direction of growth to determine softness or coarseness.

Overall Coat Condition

Overall coat condition refers to the uniformity of the fur all over the body (ignoring the head, paws, and tail). If the is a solid color, then the color intensity and shading should be considered. The length of hair should be the same all over for a uniform coat. Whether the hair stands up or lies smooth, is straight, wavy, or curly should also be considered. It should be noted that uniformity is due to genetic factors but is affected by environmental conditions as well.
